# Long non-coding RNA ARAP1-AS1 contributes to cell proliferation and migration in clear cell renal cell carcinoma via the miR-361-3p/placental growth factor axis

**DOI:** 10.1080/21655979.2021.1975019

**Published:** 2021-09-13

**Authors:** Liping Zhong, Xiuwen Zhong

**Affiliations:** a Department of Kidney Disease of Internal, Hubei Hospital of Integrated Traditional Chinese and Western Medicine, Wuhan, Hubei, China; b Department of Rehabilitation Medicine Center, Wuhan Central Hospital of Hubei Province, Wuhan, Hubei, China

**Keywords:** ARAP1-AS1, miR-361-3p, PGF, clear cell renal cell carcinoma

## Abstract

Clear cell renal cell carcinoma (ccRCC) is an aggressive malignancy with a poor prognosis. Therefore, investigating the molecular mechanism of ccRCC is important for ccRCC treatment. Here, we aimed to explore the effect of the long non-coding RNA ARAP1-AS1/miR-361-3p/PGF axis on ccRCC. The expression of lncRNA ARAP1-AS1, miR-361-3p, and placental growth factor (PGF) in ccRCC cells was verified by real-time quantitative PCR (RT-qPCR). The influence of the ARAP1-AS1/miR-361-3p/PGF axis on ccRCC cells was identified using the Cell Counting Kit-8 (CCK-8) assay, colony formation assay, flow cytometry, and wound healing assay. The interaction between ARAP1-AS1, miR-361-3p, and PGF was confirmed by bioinformatics analysis and luciferase assay. The results showed that the levels of ARAP1-AS1 and PGF increased in ccRCC cells, while miR-361-3p expression decreased. Cell functional experiments showed that cell proliferation and migration were inhibited by silencing ARAP1-AS1 or PGF, while miR-361-3p inhibitor or PGF overexpression could relieve the inhibitory effect of silencing ARAP1-AS1 on ccRCC cells. Moreover, ARAP1-AS1 sponges miR-361-3p to increase PGF expression. In conclusion, our study revealed that ARAP1-AS1 enhanced the malignancy of ccRCC cells by regulating the miR-361-3p/PGF axis.

## Introduction

Clear cell renal cell carcinoma (ccRCC), which accounts for approximately 70% of renal cell carcinomas, is a common pathological type of renal cell carcinoma [1]. It has been reported that the prognosis of ccRCC is poor due to relapse, and 30% of ccRCC patients experience metastasis [2]. At present, patients diagnosed with ccRCC at early and advanced stages often undergo surgery or drug therapy [3]. For example, sunitinib and pazopanib have been used to treat advanced or metastatic ccRCC [4]. However, the prognosis of patients with ccRCC remains low owing to the limitation of drug efficacy [4,5]. Therefore, identification of novel biomarkers is crucial for the treatment of ccRCC.

Long noncoding RNAs (lncRNAs) with a length longer than 200 nucleotides have been reported to participate in the progression of multiple cancers, even though they cannot code for protein [6–9]. As for the mechanism of lncRNAs participating in cancer progression, lncRNAs can regulate gene expression by splicing regulation, epigenetic silencing, or sponging miRNAs [10–12]. In ccRCC, lncRNA URRCC was found to enhance the growth and invasion of ccRCC cells and was closely related to the poor prognosis of ccRCC [13]. Hu *et al*. proved that lncRNA MSC-AS1 contributed to cell proliferation and migration in ccRCC by sponging miR-3924/WNT5A [14]. lncRNA ARAP1-AS1 (ARAP1 antisense RNA 1), a member of the lncRNA family, has been shown to be a tumor promoter in multiple cancers such as bladder [15], cervical [16], and breast cancer [17]. However, the effect of ARAP1-AS1 on ccRCC has not yet been explored.

MicroRNAs (miRNAs) act as key regulators in the development of cancers by inhibiting the expression of their target genes [18–20]. miR-361-3p, an miRNA, has been confirmed as an anti-tumor factor in non-small cell lung cancer [21], cervical cancer [22], and thyroid cancer [23]. A study showed that miR-361-3p sponged by lncRNA BBOX1-AS1 inhibited colorectal cancer progression by targeting SH2B adaptor protein 1 [24]. Sun *et al*. [25] also found that miR-361-3p inhibited by circ_0000034 could prevent the progression of retinoblastoma. These studies suggested that miR-361-3p could be sponged by lncRNA or circRNA to participate in cancer progression. Although miR-361-3p has not been investigated in ccRCC, our bioinformatics analysis revealed that miR-361-3p could bind to ARAP1-AS1 and PGF, thereby playing a key role in ccRCC.

Placental growth factor (PGF), also called PIGF, a member of the vascular endothelial growth factor (VEGF) sub-family, was originally discovered in the human placenta [26]. Recently, PGF has been reported to be a key mediator of tumor angiogenesis in various cancers [27]. Aberrant expression has been reported in multiple cancer types, such as gastric [28], breast [29], and colorectal cancer [30]. For instance, PGF could promote tumor growth in non-small cell lung cancer by triggering macrophage polarization to a tumor-associated macrophage subtype [31]. In ccRCC, PGF was shown to be upregulated in ccRCC serum samples, and its high expression suggested the poor prognosis of ccRCC [32]. However, the regulatory mechanism of PGF involving the key lncRNAs and miRNAs upstream remains unclear.

In our study, we investigated the roles of ARAP1-AS1, miR-361-3p, and PGF in ccRCC using bioinformatics analysis. We aimed to identify the effect of the ARAP1-AS1/miR-361-3p/PGF axis in ccRCC by cell functional experiments. Our findings help in revealing the molecular mechanisms involved in ccRCC and identifying biomarkers for diagnosis and therapy.

## Material and methods

### Tissues and cell lines

The ccRCC tissues and adjacent non-carcinoma tissues were collected from 16 patients diagnosed with ccRCC at the Hubei Hospital of Integrated Traditional Chinese and Western Medicine between June 2019 and December 2020. All samples were diagnosed by three independent pathologists and stored in liquid nitrogen. Informed consent forms were signed by 16 ccRCC patients, and our study was approved by the Ethical Committee of Hubei Hospital of Integrated Traditional Chinese and Western Medicine. The clinical characteristics of 16 patients with ccRCC are shown in Supplementary Table I.

The human normal renal tubular epithelial cell line (HK-2) and human ccRCC cell lines (Caki-1 and A498) were provided by the BeNa Culture Collection (BNCC, China). HK-2 cells were cultured in Dulbecco’s modified Eagle’s medium (DMEM)-H medium (Gibco, USA), Caki-1 cells were cultured in McCoy’s 5a medium (Gibco), and A498 cells were cultured in Roswell Park Memorial Institute (RPMI)-1640 medium (Gibco). All cells were cultured with 10% fetal bovine serum (FBS, Gibco) in an incubator at 37°C and 5% CO_2_.

### Real-time quantitative PCR (RT-qPCR)

Total RNA was isolated using TRIzol (Thermo Scientific, USA), following the manufacturer’s protocol, and quantified using a NanoDrop 2000 (Thermo Scientific, USA). Then, the reverse transcription kit (Takara, Japan) was used to reverse transcribe RNA to cDNA, and SYBR green mix (Takara, Japan) was used to perform RT-qPCR with an ABI7500 quantitative PCR instrument (Applied Biosystems. USA). The lncRNAs and mRNAs were normalized to GAPDH, and miRNA was normalized to U6. The 2^−ΔΔCt^ method [33] was used to calculate relative expression. All primers used for RT-qPCR are listed in Supplementary Table II.

### Cell transfection

siRNAs for ARAP1-AS1 (si-ARAP1-AS1), miR-361-3p inhibitor, miR-361-3p mimic, and their corresponding negative controls (NC) including si-NC, inhibitor-NC, and mimic-NC were purchased from RiboBio (China). The PGF overexpression vectors (pcDNA3.1-PGF) were also purchased from RiboBio, and pcDNA 3.1 empty vectors were used as NC of pcDNA3.41-PGF. Lipofectamine 3000 (Invitrogen) was used for cell transfection. Briefly, 2 × 10^6^ cells were seeded in 6-well plates, incubated overnight, and transfected with 50 nM si-ARAP1-AS1, 50 nM miR-361-3p inhibitor, 50 nM si-NC, 50 nM inhibitor-NC, 50 nM pcDNA3.1, and 50 nM pcDNA3.1-PGF using Lipofectamine 3000. After 48 h, the transfection efficiency was determined by RT-qPCR.

### Cell proliferation assay

Cell proliferation was detected using the Cell Counting Kit 8 (CCK-8) provided by Dojindo (Japan) [34]. After transfection, the cells were seeded in 96-well plates at a density of 1500 cells/well. Cell proliferation was detected at an interval of 24 h by adding 10 μL CCK-8 for 2 h incubation. Finally, cell proliferation was determined by measuring the absorbance at 450 nm using a microplate reader (Thermo Scientific, USA).

### Colony formation assay

The colony formation assay was performed according to a previously described method [35]. After transfection, 500 cells/well were plated in 6-well plates in medium supplemented with 10% FBS. After incubation for two weeks, the colonies were fixed with 4% paraformaldehyde and incubated with crystal violet (Sigma-Aldrich, China) for 15 min. Images of colony formation were captured under an inverted microscope.

### Cell apoptosis by flow cytometry

Cell apoptosis was detected using the Annexin V-FITC cell apoptosis kit (Beyotime, China) according to a previously described method [36]. After transfection, the cells were collected and washed with PBS. Then, 200 μL binding buffer containing 5 μL Annexin V-FITC and 10 μL PI was applied for 20 min incubation without light. Finally, the apoptosis rate was assessed using flow cytometry (Becton Dickinson, USA).

### Wound healing assay

Cell migration was assessed using a wound healing assay, as previously described [37]. After transfection, 2 × 10^6^ cells were seeded in 6-well plates and incubated until 90% confluence. Then, 200 μL pipette tips were used to scratch the wound, and the exfoliated cells were removed with PBS. Fresh serum-free medium was added to the cells and incubated for 24 h. Images of cells migrating into the wound surface were captured using an inverted microscope at 0 and 24 h.

### Luciferase assay

The luciferase assay was performed according to a previously described method [38]. Based on the predicted binding sites between ARAP1-AS1/PGF and miR-361-3p by miRDB and TargetScan, wild-type (WT)-ARAP1-AS1, mutant (MUT)-ARAP1-AS1, WT-PGF, and MUT-PGF were designed and constructed using the pGL3-REPORT luciferase reporter vector (Promega, USA) by RiboBio (China). Then, the cells were seeded into 24-well plates and co-transfected with WT or MUT vectors and mimic-NC or miR-361-3p mimic for 48 h. Finally, luciferase activity was detected using the Dual-Luciferase Assay System (Promega, USA).

### RNA pull-down assay

After reaching >80% confluence, the cells were treated with 500 μL mixture containing 25 mM Tris-HCl, 70 mM KCl, 0.05% NP-40, 80 U/mL RNase inhibitor, and 2.5 mM EDTA. After centrifuging at 12,000 x *g* for 15 min, biotin-labeled miR-361-3p mimic (bio-mimic) or mimic-NC (bio-NC) from RiboBio (China) was added to the supernatant and incubated for 30 min. Then, 10 μL Streptavidin Mutein Matrix (Roche Applied Science) was added to the supernatant and incubated for 3 h. Finally, the mixture was collected and washed five times to obtain the biotin-miRNA/mRNA complex. RT-qPCR was used to detect bound RNA expression [39].

### Statistical analysis

Data from three independent experiments are presented as the mean ± S.D. Statistical analysis was performed using GraphPad Prism 6. The differences were compared by paired Student’s t-test for two groups, and one-way or two-way analysis of variance (ANOVA) following Dunnett’s or Tukey’s multiple comparisons for multiple groups. Pearson correlation analysis was used to analyze the relationship between ARAP1-AS1, miR-361-3p, and PGF in ccRCC tissues. Statistical significance was set at *P*< 0.05.

## Results

Based on bioinformatics analysis and literature review, we suspected that ARAP1-AS1, miR-361-3p, and PGF might play key roles in ccRCC. Therefore, in this study, we aimed to reveal the functions of ARAP1-AS1, miR-361-3p, and PGF in ccRCC. The results showed the positive roles of ARAP1-AS1 and PGF, and the negative role of miR-361-3p in ccRCC by regulating proliferation, migration, and apoptosis. Our findings provide a new therapeutic target for ccRCC treatment.

### ARAP1-AS1 was the key regulator in ccRCC

**Figure 1.**
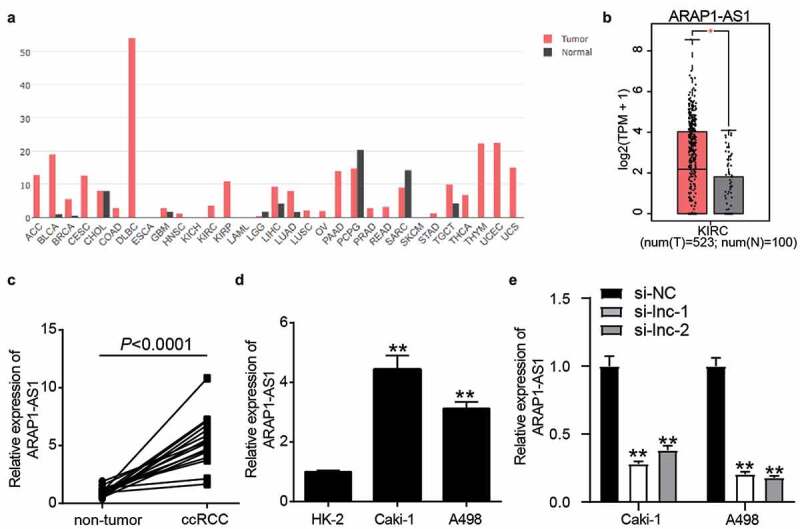
ARAP1-AS1 plays the key role in ccRCC. (a) The expression of ARAP1-AS1 in multiple cancers based on GEPIA analysis. (b) The expression of ARAP1-AS1 in ccRCC and normal samples based on GEPIA analysis. *, P < 0.01. (c) The expression of ARAP1-AS1 in ccRCC and non-tumor tissues from 16 patients diagnosed with ccRCC in our hospital. (d) The expression of ARAP1-AS1 in human normal renal tubular epithelial cell line (HK-2) and human ccRCC cell lines (Caki-1 and A498) by RT-qPCR. **, P < 0.001 compared with HK-2. (e) The transfection efficiency of siRNAs targeting to ARAP1-AS1 was identified by RT-qPCR. NC, negative control. si-lnc-1 and si-lnc-2 were two siRNAs of ARAP1-AS1. **, P < 0.01 compared with si-NC

Based on gene expression profiling interactive analysis (GEPIA), ARAP1-AS1 was upregulated in multiple cancers and significantly increased in ccRCC samples (Figure 1a and Figure 1b). In our collected clinical samples, ARAP1-AS1 was upregulated 5-fold in ccRCC samples compared to that in non-tumor samples (Figure 1c). After dividing the clinical samples into high- and low-expression groups based on the mean expression of ARAP1-AS1, it was found that ARAP1-AS1 expression was closely related to T stage and distant metastasis (Supplementary Table I). Compared with HK-2 cells, ARAP1-AS1 expression increased in Caki-1 cells by 4.3-fold and in A498 cells by 3.6-fold (Figure 1d). After transfecting two siRNAs of ARAP1-AS1 into ccRCC cells, it was found that two siRNAs of ARAP1-AS1 significantly reduced ARAP1-AS1 expression (Figure 1e). Our results suggest that ARAP1-AS1 may be a key regulator of ccRCC.

### ARAP1-AS1 silence suppressed the malignancy of ccRCC cells

**Figure 2.**
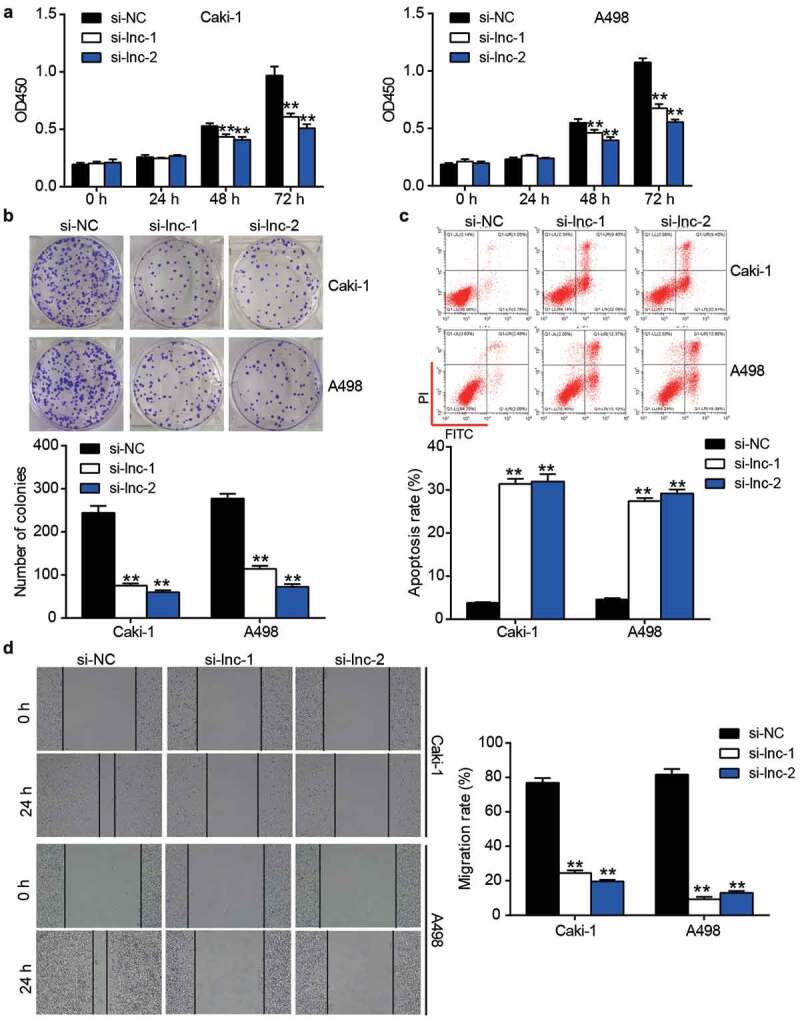
The negative effect of ARAP1-AS1 knockdown on ccRCC cells. (a) The cell proliferation was detected by CCK-8 assay in ccRCC cells with the transfection of si-ARAP1-AS1. (b) The colony formation ability was identified by colony formation assay in ccRCC cells with the transfection of si-ARAP1-AS1. (c) The cell apoptosis was measured by flow cytometry in ccRCC cells with the transfection of si-ARAP1-AS1. (d) The cell migration was verified by wound healing assay in ccRCC cells with the transfection of si-ARAP1-AS1. NC, negative control. si-lnc-1 and si-lnc-2 were two siRNAs of ARAP1-AS1. **, P < 0.01 compared with si-NC.+

After performing the CCK-8 assay, it was found that si-ARAP1-AS1 impaired cell proliferation in Caki-1 and A498 cells after transfection for 48 and 72 h (Figure 2a). The colony formation assay further proved that silencing ARAP1-AS1 inhibited cell proliferation (Figure 2b). For cell apoptosis, the results from flow cytometry showed that silencing ARAP1-AS1 induced cell apoptosis in Caki-1 and A498 cells (Figure 2c). In addition, the wound healing assay confirmed that cell migration was inhibited in the si-ARAP1-AS1 groups (Figure 2d). These cell functional experiments suggested that downregulation of ARAP1-AS1 could suppress the malignancy of ccRCC cells.

### miR-361-3p/PGF axis might be the downstream of ARAP1-AS1 in ccRCC

**Figure 3.**
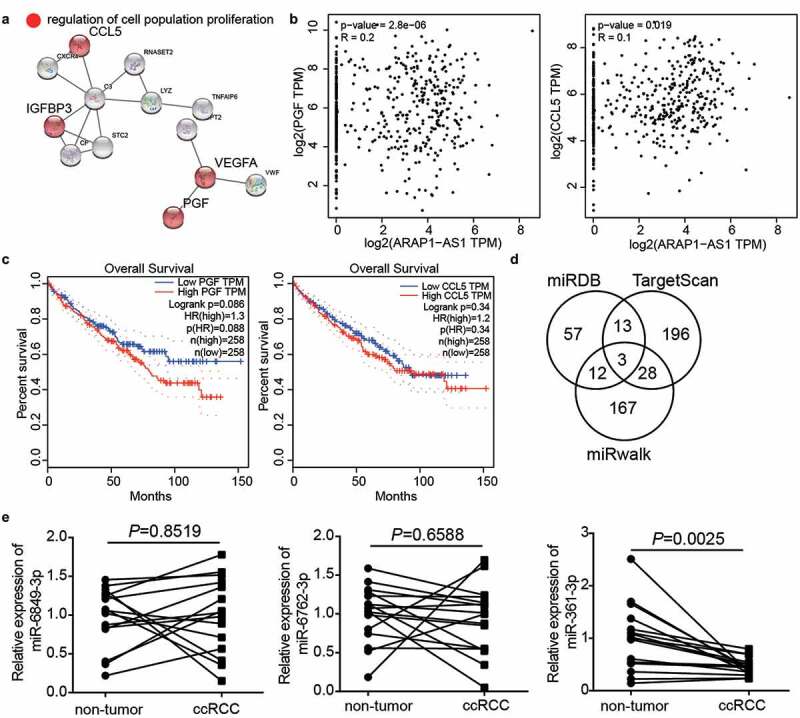
miR-361-3p/PGF might be the downstream of ARAP1-AS1 by bioinformatics analysis. (a) CCL5, IGFBP3, VEGFA and PGF were predicted to be related to cell proliferation by STRING analysis. (b) PGF and CCL5 were positive correlated with ARAP1-AS1 in ccRCC samples based on GEPIA analysis. (c) PGF with high expression showed the poor prognosis of ccRCC by GEPIA analysis. (d) Three miRNAs were overlapped from miRDB, TargetScan, and miRwalk. miRDB was used to predict miRNAs sponged by ARAP1-AS1. TargetScan and miRwalk were used to predict the miRNAs targeting PGF. (e) The miR-361-3p expression reduced in ccRCC samples

With adj.P < 0.01 and log_2_FC>3, 72 upregulated genes were screened in ccRCC samples based on GEPIA analysis. Uploading 72 upregulated genes to STRING for GO enrichment resulted in the identification of the involvement of regulation of cell population proliferation including C-C motif chemokine ligand 5 (CCL5), vascular endothelial growth factor A (VEGFA), PGF, and insulin like growth factor binding protein 3 (IGFBP3) (Figure 3a). GEPIA analysis revealed that the expression of PGF and CCL5 was positively related to ARAP1-AS1 expression in ccRCC samples (Figure 3b). The prognosis of ccRCC from GEPIA showed that PGF expression was closely associated with prognosis compared with that of CCL5 (Figure 3c), confirming that PGF was our gene of interest. To explore the miRNA connecting PGF and ARAP1-AS1, miRDB was used to predict miRNAs sponged by ARAP1-AS1, whereas TargetScan and miRwalk were used to predict miRNAs targeting PGF. The results showed that three miRNAs, miR-6849-3p, miR-6762-3p, and miR-361-3p, were the common miRNAs in miRDB, TargetScan, and miRwalk (Figure 3d). Because of the low expression of miR-361-3p in ccRCC tissues (Figure 3e) and the close relationship between miR-361-3p expression and T stage (Supplementary Table I), miR-361-3p was identified as an interesting miRNA. Therefore, the miR-361-3p/PGF axis was identified as the downstream target of ARAP1-AS1 in ccRCC.

### miR-361-3p could be sponged by ARAP1-AS1 in ccRCC cells

**Figure 4.**
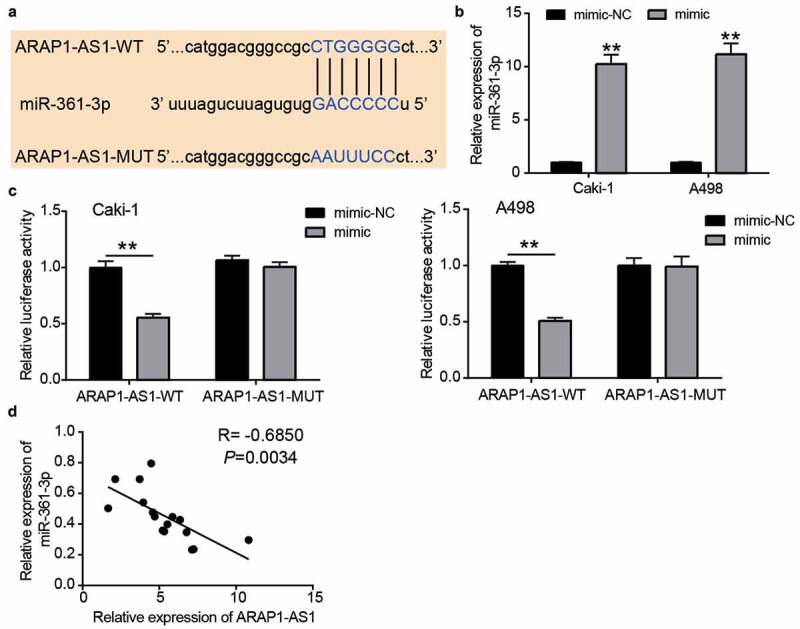
miR-361-3p could be sponged by ARAP1-AS1. (a) The binding sites were predicted by miRDB. (b) miR-361-3p mimic upregulated miR-361-3p expression in Caki-1 and A498 cells. NC, negative control. mimic, miR-361-3p mimic. **, P < 0.01 compared with mimic-NC. (c) Luciferase assay proved the targeting relationship between ARAP1-AS1 and miR-361-3p. WT, wild-type. MUT, mutant. NC, negative control. mimic, miR-361-3p mimic. **, P < 0.01. (d) miR-361-3p expression was negative related to ARAP1-AS1 expression in ccRCC tissues

The sequence of the wild-type or mutant binding sites is shown in Figure 4a. To verify this relationship, the miR-361-3p mimic was first transfected into Caki-1 and A498 cells (Figure 4b). The luciferase assay showed that the miR-361-3p mimic reduced the luciferase activity in the ARAP1-AS1-WT group, whereas it did not change the luciferase activity in the ARAP1-AS1-MUT group (Figure 4c). In addition, miR-361-3p expression was negatively correlated with ARAP1-AS1 expression in ccRCC tissues (R = −0.6850, Figure 4d). These data showed that miR-361-3p could be sponged by ARAP1-AS1 in ccRCC cells.

### miR-361-3p inhibitor relieved the inhibitory effect of si-ARAP1-AS1 on ccRCC cells

**Figure 5.**
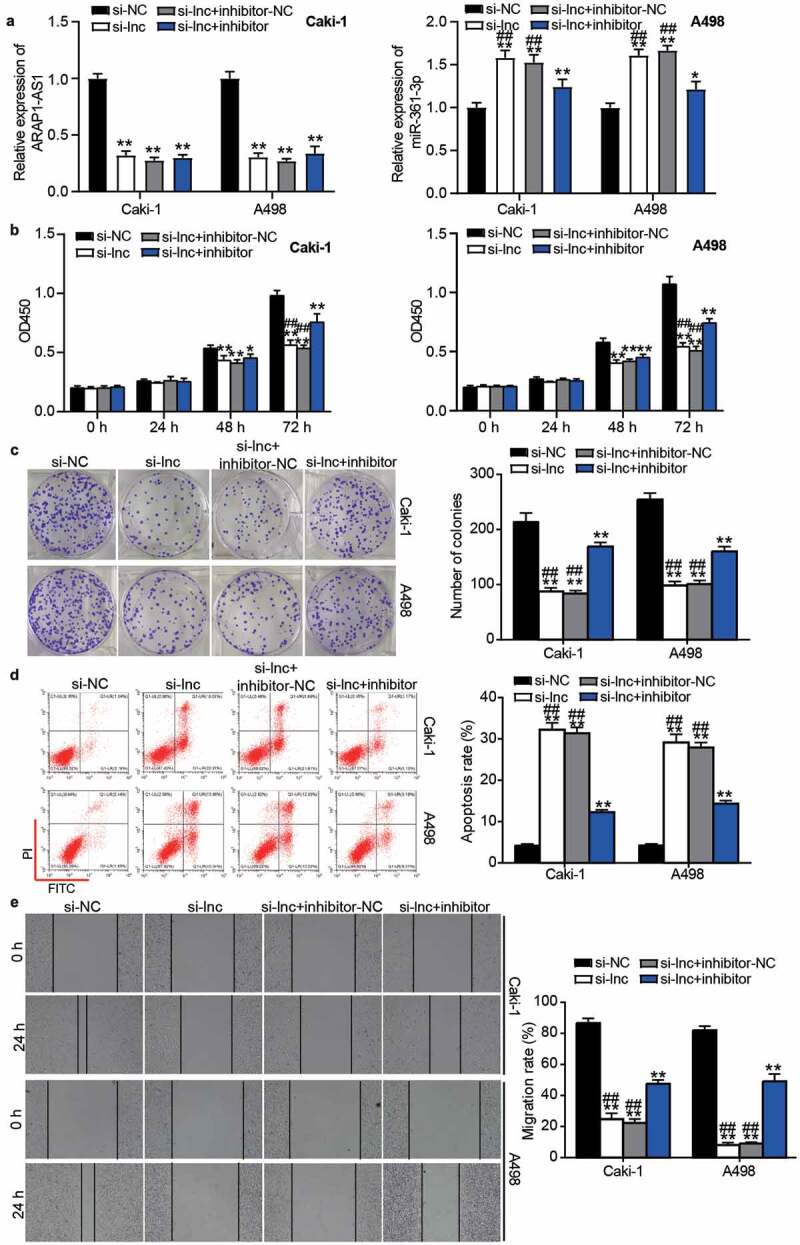
The negative effect of si-ARAP1-AS1 on ccRCC cells was relieved by miR-361-3p inhibitor. (a) The expression of ARAP1-AS1 and miR-361-3p in transfected ccRCC cells. (b) The cell proliferation was detected by CCK-8 assay in transfected ccRCC cells. (c) The colony formation ability was identified by colony formation assay in transfected ccRCC cells. (d) The cell apoptosis was measured by flow cytometry in transfected ccRCC cells. (e) The cell migration was verified by wound healing assay in transfected ccRCC cells. NC, negative control. si-lnc, siRNA of ARAP1-AS1. inhibitor, miR-361-3p inhibitor. *, P < 0.05 and **, P < 0.01 compared with si-NC. ##, P < 0.01 compared with si-lnc+inhibitor

Following transfection of miR-361-3p inhibitor and si-ARAP1-AS1 into Caki-1 and A498 cells, the results showed that ARAP1-AS1 expression was reduced, whereas miR-361-3p expression increased by 1.5-fold in the si-ARAP1-AS1 group but was reduced in the co-transfection of miR-361-3p inhibitor and si-ARAP1-AS1 group compared with the si-ARAP1-AS1 group (Figure 5a). The CCK8 and colony formation assays showed that the inhibitory effect of si-ARAP1-AS1 on cell proliferation could be relieved by the miR-361-3p inhibitor (Figure 5b and Figure 5c). For cell apoptosis, the enhanced apoptosis rate in the si-ARAP1-AS1 group was decreased in the cells co-transfected with si-ARAP1-AS1 and miR-361-3p inhibitor (Figure 5d). In addition, the miR-361-3p inhibitor also partly revised the negative effect of si-ARAP1-AS1 on cell migration (Figure 5e). These findings indicate that ARAP1-AS1 regulates the malignancy of ccRCC cells by sponging miR-361-3p.

### PGF was a target of miR-361-3p

**Figure 6.**
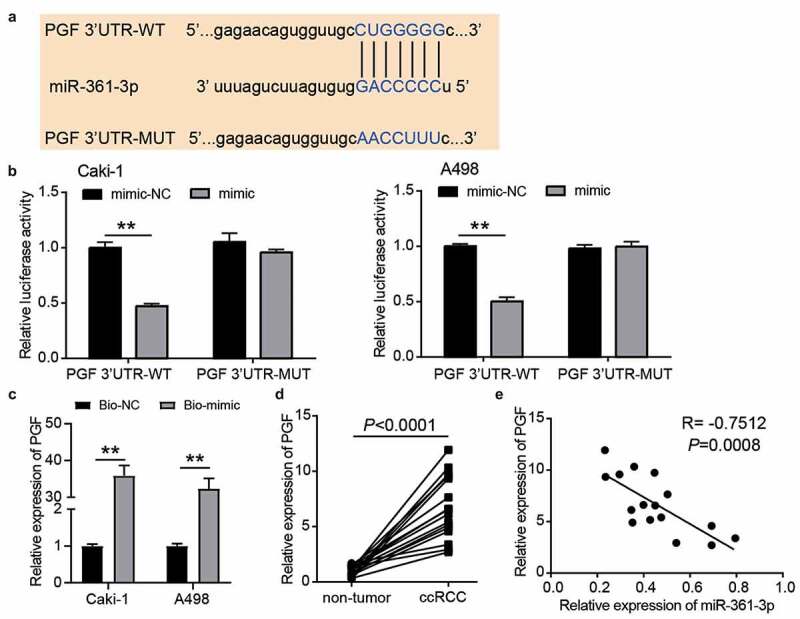
PGF was the target of miR-361-3p in ccRCC cells. (a) The binding sites were predicted by TargetScan. (b) Luciferase assay proved the targeting relationship between PGF and miR-361-3p. WT, wild-type. MUT, mutant. NC, negative control. mimic, miR-361-3p mimic. **, P < 0.01. (c) RNA pull-down assay confirmed the direct interaction between miR-361-3p and PGF. **, P < 0.01. Bio-NC, biotin-labeled negative control. Bio-mimic, biotin-labeled miR-361-3p mimic. (d) The PGF expression reduced in ccRCC tissues. (e) PGF expression was negative related to miR-361-3p expression in ccRCC tissues

According to the prediction from TargetScan, the wild-type and mutant binding sites between PGF and miR-361-3p are shown in Figure 6a. The luciferase assay showed that luciferase activity was reduced when Caki-1 and A498 cells were co-transfected with PGF 3ʹUTR-WT and miR-361-3p mimic (Figure 6b). After performing the RNA pull-down assay, we found that PGF was enriched in the miR-361-3p mimic group, suggesting that miR-361-3p could bind to PGF (Figure 6c). In our collected tissue samples, PGF expression increased 7-fold in ccRCC samples (Figure 6d), and its expression was related to T stage (Supplementary Table I). In addition, PGF expression was negatively correlated with miR-361-3p expression in ccRCC samples (R = −0.7512, Figure 6e). These results proved that PGF was downstream of miR-361-3p in ccRCC cells.

### PGF overexpression partly relieved the inhibitory effect of si-ARAP1-AS1 on ccRCC cells

**Figure 7.**
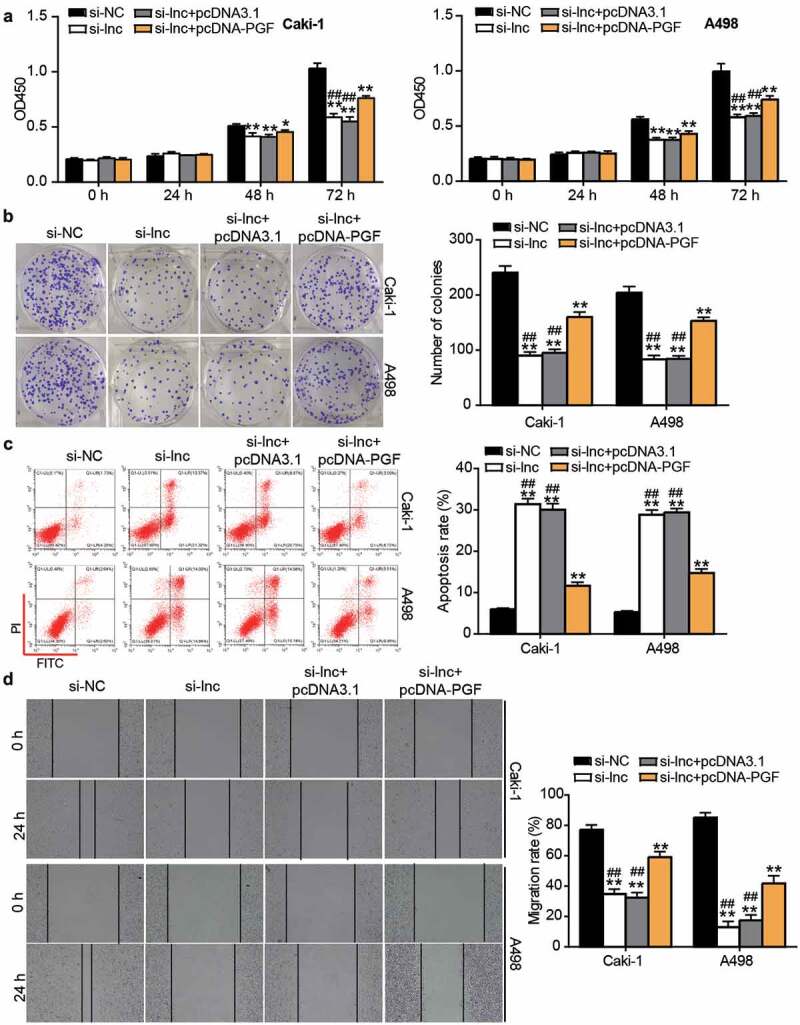
The negative effect of si-ARAP1-AS1 on ccRCC cells was relieved by PGF overexpression vectors. (a) The cell proliferation was detected by CCK-8 assay in transfected ccRCC cells. (b) The colony formation ability was identified by colony formation assay in transfected ccRCC cells. (c) The cell apoptosis was measured by flow cytometry in transfected ccRCC cells. (d) The cell migration was verified by wound healing assay in transfected ccRCC cells. NC, negative control. si-lnc, siRNA of ARAP1-AS1. pcDNA-PGF, PGF overexpression vectors. *, P < 0.05 and **, P < 0.01 compared with si-NC. ##, P < 0.01 compared with si-lnc+pcDNA-PGF

After performing the CCK8 assay, it was found that cell proliferation was enhanced by co-transfecting si-ARAP1-AS1 and PGF overexpression vectors (pcDNA-PGF) compared with the si-ARAP1-AS1 group (Figure 7a). Similar to the CCK8 assay, the colony formation assay further proved that the inhibitory effect on cell proliferation caused by si-ARAP1-AS1 was enhanced by co-transfection with pcDNA-PGF (Figure 7b). Flow cytometry showed that the apoptosis rate was reduced by co-transfection in the si-ARAP1-AS1 and pcDNA-PGF group compared with the si-ARAP1-AS1 group (Figure 7c). In addition, the wound healing assay revealed that PGF overexpression upregulated the low migration rate caused by si-ARAP1-AS1 (Figure 7d). The data indicated that PGF, a downstream target of miR-361-3p, could affect the effect of ARAP1-AS1 on ccRCC cells.

## Discussion

ccRCC, an aggressive malignancy that occurs worldwide, was investigated to reveal its mechanism and improve its poor prognosis. Recently, numerous studies have uncovered that the alteration of oncogenes and anti-tumor genes can regulate ccRCC progression [40–42]. However, the precise regulatory mechanisms remain unclear. In our study, we revealed that ARAP1-AS1 enhanced ccRCC cell proliferation and migration and impaired cell apoptosis. We also proved that ARAP1-AS1 regulates ccRCC progression by inhibiting miR-361-3p to enhance PGF expression.

Accumulating evidence has shown that lncRNAs are key regulators in ccRCC. For instance, lncRNA DLEU1 was reported to increase in ccRCC and contribute to cell proliferation by regulating miR-194-5p [43]. Here, we found that lncRNA ARAP1-AS1 was also upregulated in ccRCC cells and was related to the malignancy of ccRCC cells. Remarkably, ARAP1-AS1 with high expression was confirmed in multiple cancers, and it could promote tumor progression, such as in cervical [44], bladder [15], and breast cancer [17]. These previous studies suggest that ARAP1-AS1 may be a tumor promoter in cancer, although it has not been explored in ccRCC. Consistent with a previous study on the effect of ARAP1-AS1 on other cancers, our results confirmed that the upregulation of ARAP1-AS1 contributed to cell proliferation, migration, and colony formation, but inhibited cell apoptosis in ccRCC cells. Our results indicate that ARAP1-AS1 may be a promising biomarker for ccRCC.

The regulatory mechanism of lncRNAs in cancers is complex and includes splicing regulation, epigenetic silencing, and sponging miRNAs [10–12]. As for sponging miRNAs, lncRNAs act as miRNA sponges to inhibit miRNA expression and upregulate the genes targeted by miRNA [45]. ARAP1-AS1 was reported to sponge miR-4735-3p and upregulate the target gene (NOTCH2) of miR-4735-3p, thereby promoting bladder cancer progression [15]. In our study, we found that ARAP1-AS1 sponging miR-361-3p upregulated PGF and played a positive role in ccRCC cells. miR-361-3p has been confirmed to play a negative role in multiple cancers [21,23,46], although its effect has not been explored in ccRCC. We proved that miR-361-3p is an anti-tumor miRNA in ccRCC by targeting PGF.

PGF found in the placenta is homologous to VEGF, but it has been reported to be a useful biomarker in the diagnosis and prediction of prognosis in ccRCC [32]. Bessho *et al*. found that TB403, a monoclonal antibody against PGF, could inhibit the VEGF pathway to regulate angiogenesis escape in ccRCC, suggesting that PGF might contribute to the development of ccRCC [47]. The mechanism of PGF involving upstream regulators in ccRCC has not yet been explored. In this study, we revealed that PGF overexpression could relieve the inhibitory effect of ARAP1-AS1 knockdown on ccRCC cells by regulating cell proliferation, migration, and apoptosis, suggesting that PGF overexpression could promote ccRCC progression. Further investigation revealed that ARAP1-AS1 and miR-361-3p were the upstream regulators of PGF, and the positive effect of PGF on ccRCC could be regulated by the ARAP1-AS1/miR-361-3p axis.

Our study revealed that ARAP1-AS1 contributes to cell proliferation and migration in ccRCC cells by sponging the miR-361-3p/PGF axis, but its effect and mechanism on ccRCC *in vivo* have not been explored because of the limitations of experimental conditions. At the same time, PGF was reported to be associated with the activation of the SAPK pathway, JNK and p38 kinase, and ERK pathway in primary human trophoblasts [48]. Whether these pathways could be regulated by PGF in ccRCC to enrich the downstream of ARAP1-AS1/miR-361-3p/PGF should be further explored. In addition, ARAP1-AS1 has been shown to participate in multiple human cancer types by sponging different miRNAs, including miR-2110 [17], miR-4735-3p [15], and miR-4735-3p [49]. Therefore, ARAP1-AS1 may sponge other miRNAs to promote the proliferation and migration of ccRCC cells, which needs to be further confirmed in the future.

## Conclusion

Our study is the first to reveal the effect of the ARAP1-AS1/miR-361-3p/PGF axis in ccRCC cells. Specifically, ARAP1-AS1 contributed to the malignancy of ccRCC cells by sponging miR-361-3p to upregulate PGF. Our research provides novel biomarkers for the diagnosis and treatment of ccRCC.

## Supplementary Material

Supplemental Material

## Data Availability

The data used to support the findings of this study are available from the corresponding author upon request.

## Highlights

1. ARAP1-AS1 expression is upregulated in clear cell renal cell carcinoma (ccRCC).

2. ARAP1-AS1 promoted cell proliferation and cell migration in ccRCC.

3. ARAP1-AS1 facilitated the malignancy of ccRCC cells by sponging the miR-361-3p/PGF axis.
