# Supplementary material for: Long non-coding RNA ARAP1-AS1 contributes to cell proliferation and migration in clear cell renal cell carcinoma via the miR-361-3p/placental growth factor axis
Source: Bioengineered. 2021 Sep 13;12(1):6629–42. doi: 10.1080/21655979.2021.1975019 (PMC8806691; doi:10.1080/21655979.2021.1975019)
Supplement: Supplemental Material [file KBIE_A_1975019_SM2683.zip › supplementary/Supplementary table II_revised.docx]

**Supplementary table II** The sequences of the primers used in this study

| **Primer** | **Sequences** |
| --- | --- |
| **ARAP1-AS1** | Forward: 5'-CCCGTGACAGTGAAACATTG-3' |
|  | Reverse: 5'-TGAATCAGCAGCTTCCACAC-3' |
| **miR-361-3p** | Forward: 5'-GCCGCTCCCCCAGGTGTGATT-3' |
|  | Reverse: 5'-GTGCAGGGTCCGAGGT-3' |
| **PGF** | Forward: 5'-CGGCGATGAGAATCTGCACT-3' |
|  | Reverse: 5'-GTGGCAGTCTGTGGGTCTC-3' |
| **GAPDH** | Forward: 5'-CAGCCTCAAGATCATCAGCA-3' |
|  | Reverse: 5'-GGCATGGACTGTGGTCATGAG-3' |
| **U6** | Forward: 5'-CTCGCTTCGGCACA-3' |
|  | Reverse: 5'-AACGCTTCACGAATTTGCGT-3' |
