# Supplementary material for: Long non-coding RNA ARAP1-AS1 contributes to cell proliferation and migration in clear cell renal cell carcinoma via the miR-361-3p/placental growth factor axis
Source: Bioengineered. 2021 Sep 13;12(1):6629–42. doi: 10.1080/21655979.2021.1975019 (PMC8806691; doi:10.1080/21655979.2021.1975019)
Supplement: Supplemental Material [file KBIE_A_1975019_SM2683.zip › supplementary/Supplementary table I_revised.docx]

**Supplementary table I** The characteristics of 16 patients with ccRCC used in this study

| Characteristics | Total=16 | ARAP1-AS1 expression | | p-value | miR-361-3p expression | | p-value | PGF expression | | p-value |
| --- | --- | --- | --- | --- | --- | --- | --- | --- | --- | --- |
|  |  | low | high |  | low | high |  | low | high |  |
| Age (years) |  |  |  | 0.6193 |  |  | 0.9999 |  |  | 0.6193 |
| >60 | 8 | 3 | 5 |  | 4 | 4 |  | 3 | 5 |  |
| ≤60 | 8 | 5 | 3 |  | 4 | 4 |  | 5 | 3 |  |
| Gender |  |  |  | 0.9999 |  |  | 0.9999 |  |  | 0.9999 |
| Male | 9 | 5 | 4 |  | 4 | 5 |  | 4 | 5 |  |
| Female | 7 | 3 | 4 |  | 4 | 3 |  | 4 | 3 |  |
| T stage |  |  |  | 0.0103 |  |  | 0.0262 |  |  | 0.0138 |
| T1 | 5 | 5 | 0 |  | 0 | 5 |  | 5 | 0 |  |
| T2 | 7 | 3 | 4 |  | 5 | 2 |  | 1 | 6 |  |
| T3 | 4 | 0 | 4 |  | 3 | 1 |  | 2 | 2 |  |
| Distant metastasis | |  |  | 0.0406 |  |  | 0.3147 |  |  | 0.3147 |
| M0 | 9 | 7 | 2 |  | 3 | 6 |  | 5 | 3 |  |
| M1 | 7 | 1 | 6 |  | 5 | 2 |  | 2 | 5 |  |

The p-value was analyzed by Fisher’s exact test.
